# Environmental surveillance of poliovirus and non-polio enterovirus in urban sewage in Dakar, Senegal (2007-2013)

**DOI:** 10.11604/pamj.2014.19.243.3538

**Published:** 2014-11-04

**Authors:** Abdou Kader Ndiaye, Pape Amadou Mbathio Diop, Ousmane Madiagne Diop

**Affiliations:** 1 National Reference Center for Enteric Viruses, Medical Virology Unit, Institut Pasteur de Dakar, Senegal

**Keywords:** Enteroviruses, poliovirus, sewage

## Abstract

**Introduction:**

Global poliomyelitis eradication initiative relies on (i) laboratory based surveillance of acute flaccid surveillance (AFP) to monitor the circulation of wild poliovirus in a population, and (ii) vaccination to prevent its diffusion. However, as poliovirus can survive in the environment namely in sewage, environmental surveillance (ES) is of growing importance as the eradication target is close. This study aimed to assess polioviruses and non polio enteroviruses circulation in sewage drains covering a significant population of Dakar.

**Methods:**

From April 2007 to May 2013, 271 specimens of raw sewage were collected using the grab method in 6 neighborhoods of Dakar. Samples were processed to extract and concentrate viruses using polyethylene glycol and Dextran (two-phase separation method). Isolation of enteroviruses was attempted in RD, L20B and Hep2 cell lines. Polioviruses were identified by RT-PCR and Elisa. Non Polio Enteroviruses (NPEVs) were identified by RT-PCR and microneutralisation tests.

**Results:**

Polioviruses and NPEVs were respectively detected in 34,3% and 42,8% sewage samples. No wild poliovirus neither circulating vaccine-derived Poliovirus (cVDPV) was detected. Neutralization assays have identified 49 non polio enteroviruses that were subsequently classified in 13 serotypes belonging to HEV-A (22, 4%), HEV-B (12, 24%), HEV-C (26, 53%) and HEV-D (6, 12%) species.

**Conclusion:**

This study is the first documentation of enteroviruses environmental detection in Senegal. It shows the usefulness of environmental surveillance for indirect monitoring of the circulation and distribution of enteroviruses in the community.

## Introduction

Human enteroviruses (HEVs) are ubiquitous, infecting ≈1 billion persons worldwide usually by fecal-oral route [1, 2]. They are associated with a wide spectrum of acute diseases from minor febrile illness to severe ones (aseptic meningitis, encephalitis, paralysis, myocarditis, neonatal enteroviral sepsis, etc). Enteroviruses could be related to chronic human disease like diabetes type 1 or cardiomyopathy [3, 4]. The risk of infection is directly correlated with poor hygiene and sanitation and overcrowding especially among population with bad quality of immunization. HEVs are classified in 4 species (HEV-A, HEV-B, HEV-C and HEV-D) covering more than 100 serotypes. Polioviruses (with 3 serotypes named PV1, PV2, and PV3) belong to HEV-C [5]. In 1988, WHO launched the Global Poliomyelitis Eradication Initiative based on effective immunization of children with efficient polio vaccines and monitoring of wild poliovirus (WPV) transmission through surveillance of Acute Flaccid Paralysis among children less than 15 years. The program mainly relied on mass immunization with the trivalent oral poliomyelitis vaccine. In 1998, paralytic poliomyelitis was endemic in more than 125 countries in 5 continents and was affecting more than a thousand children every day. In 2013, only three countries remain endemic for indigenous transmission of WPV (Afghanistan, Nigeria, and Pakistan) and the number of cases declined by more than 99% (from 350,000 in 1988 to 223 as of 23 October 2013 including 217 cases in endemic countries). As the Global Poliomyelitis Eradication Initiative is getting closer to the target date of 2015, timeliness and accuracy for detecting wild poliovirus circulation in population is critical as the proportion of AFP cases caused by polioviruses will approach zero, and there will be a loss of sensitivity of AFP surveillance [6]. Notwithstanding presence or absence of clinical symptoms, the poliovirus is continually replicated in intestinal lymphatic tissue for several weeks and is excreted and shed in environment through faces. This excretion may contaminate surface water sources for drinking water, recreational activities, aquaculture and irrigation. The amount of virus so excreted can reach 107 infectious dose/day per person. Environment might lead to poliovirus reintroduction in certified “polio-free-area” especially in population groups with low immunization coverage. Oral poliovirus vaccine strains, vaccine-derived polioviruses (VDPV), and even wild-type poliovirus strains may remain infectious for as long as 2 months in sewage depending upon environmental factors (inactivation by sunlight, high temperature, etc.). Circulation of EVs in sewage is a proven indicator of their presence in given communities [7]. Environmental surveillance can provide valuable supplementary information, particularly in urban populations with bad quality of surveillance, particularly in context of virus circulation or re-introduction [8].

Several studies have demonstrated the usefulness of environmental surveillance as an additional tool to determine the epidemiology of viruses circulating in given communities [9–24]. In Nigeria, in 2012, wild poliovirus type 1(WPV1) was isolated from multiple sewage samples from Kano and Sokoto state [25]. In Egypt, WPV1 was isolated from two samples collected in Cairo in December 2012. The WPV1 sequences from these isolates were similar to WPV1 circulating in northern Sindh, Pakistan. WPV has not been detected in persons with AFP in Egypt since 2004 [26]. Israel has detected 67 wild poliovirus type 1 (WPV1) positive sewage samples from 24 sampling sites, collected from 3 February 2013 to 4 August 2013 [27–30]. WHO has confirmed an outbreak of at least ten cases of polio in Syria, where vaccination coverage has dramatically decreased during the civil war.

The World Health Organization has included environmental Poliovirus surveillance in the new Strategic Plan of the Global Polio Eradication Initiative, as a supplement to Acute Flask Paralysis (AFP) surveillance. In that perspective enhanced AFP surveillance combined with environmental surveillance of polioviruses by examining sewage samples which has been shown to be more sensitive for detection of low circulation of WPV and VDPV should be an interesting strategy to implement for eradication. Indeed many viral agents including polioviruses and non polio enteroviruses have been recovered from none or inefficiently treated sewage water. In Senegal, thousands of doses of OPV are delivered every year to control PV circulation. Hence, WPV was not detected from 1998 to 2010 when an outbreak with 18 AFP cases with WPV occurred between January and April following separate introductions from Mauritania and Guinea. Since then, no WPV was detected. We initiated sampling of sewage in selected sewage plants in Dakar as pilot study for feasibility of environmental surveillance, to detect early introduction and/or silent transmission of wild poliovirus or VDPV and then facilitate a rapid response/control.

## Methods

### Sampling sites

**Figure 1:**
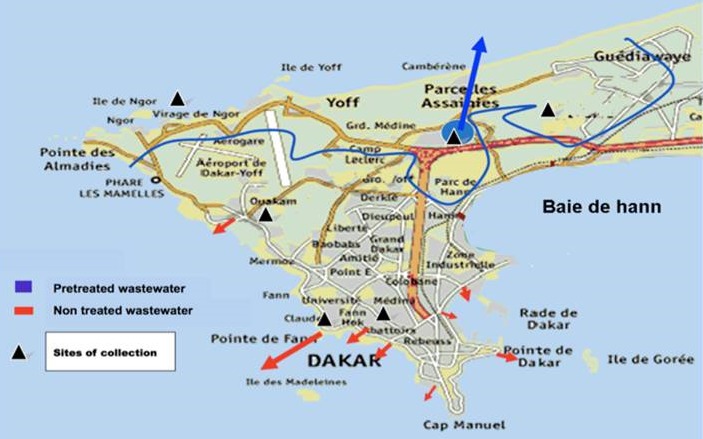
Map of Dakar indicating sampling sites from which the specimens have been collected for environmental surveillance for enteroviruses

Dakar is the westernmost city on Africa mainland and is located on the Cap-Vert Peninsula in the Atlantic Ocean. It is divided into 19 administrative areas and the population is approximately 3,000,000 (Figure 1). Six sites were selected based on size of population, socioeconomic status, accessibility, immunization coverage, sanitation, hygiene conditions and proximity with industrial areas to avoid drainage of chemical wastes. Since 1989, Dakar wastewater system is divided in 2 parts: a) The grey waters of the northern part of Dakar (concerning around 1,600,000 people) are partly drained to the sea after pre-treatment at Camberene sewage treatment plant. A portion of treated water is utilized for irrigating agricultural areas and providing the major part of vegetables sold in several markets in Dakar. b) For the Western and Southern zones of the peninsula the untreated grey water is directly drained to the sea after mechanical removal of main floating objects (rags, plastics, sticks, cans, etc.) (Figure 1).

### Sewage collection and processing

From April 2007 (Week 18) to May 2013 (Week 20) raw sewages were collected between 09.00 am and 11.00 am using the grab method as described in the WHO Guidelines for Environmental Surveillance of Poliovirus. Briefly, 1 liter of sewage (after decantation within the plant or directly in trenches) was collected using a bucket and with strict compliance to safety requirements. Sewage samples were transferred into clean and sterile labeled glass bottles, stored in a cooler and transported to the Polio Laboratory at Institut Pasteur de Dakar for processing within 1 h after collection. The concentration of wastewater samples was performed according to the method described previously by Lewis and Metcalf [31]. Briefly, the sewage sample was centrifuged at 2,500 rpm (1000 × g) for 10 min at 4°C in a centrifuge using swinging rotor. The supernatant was then collected and the pH adjusted to 7 -7.5. The pellet was stored at 4°C for further use. For each sample, 35 mg of Sodium chloride 5M, 287 ml of polyethylene glycol 6000 at 29% (i.e. 60 g per liter) and 39, 5 ml of Dextran T40 at 22% were added to 500 ml of sewage supernatant. The mixture was stirred with a magnetic stirrer for 60 min at 4°c and poured into a sterile separation funnel for each sample and left to stand overnight at +4°C to allow phase separation. The entire lower phase was harvested and the interphase collected slowly into a sterile 50ml centrifuge tube. The initially saved pellet was then resuspended into the harvested concentrate. Twenty percent chloroform was added to it, mixed thoroughly by vortexing and then centrifuged at 4°C for 20 min at 1500g. Fungizone (0,5%), Penicillin G (100 UI/ml) and Streptomycin (100 mg/ml) were added after chloroform extraction and aliquots were stored at -20°c until inoculation into cells.

### Viral isolation

Isolation of Poliovirus was carried out in low-passaged RD (rhabdomyosarcoma), L20B (cells genetically engineered to express the human poliovirus receptor [32, 33], and Hep2 (Human Caucasian larynx carcinoma epithelial cells) cell-lines. Flat-sided plastic tubes seeded with RD, L2OB, and Hep2 cells were inoculated in duplicate with 0.2ml of specimen concentrate, incubated at 36°C for 5 days. A culture was considered “positive for virus replication” when at least 75% of the cell monolayer presents CPE (cytopathic effect). All positive tubes were kept at -20°C while any culture negative in RD, L20B, and Hep2 was passaged on fresh cells and examined for 5 more days. Sample showing CPE in L20B was classified as “suspected poliovirus”. Any culture negative in L20B but positive in RD or Hep2 was classified as “non polio enterovirus”.

### Poliovirus typing and intratypic differentiation (ITD)

All cell culture positive samples collected from 2007 to 2010 were characterized using a RT-PCR kit (provided by Centers of Disease Control and Prevention, Atlanta, USA) for the ITD of polioviruses including separate reactions with pan-enterovirus and pan-poliovirus primers. Confirmed polioviruses were further characterized using serotype specific, and Sabin type 1, 2, and 3 specific primers. Amplified products were loaded on 10% polyacrylamide gels (Bio-Rad) and electrophoresed at 100 V. The PCR products were visualized under UV lights after staining for 5 min in 2 μg/ml ethidium bromide. Cell culture positive samples collected from 2011 to 2013 were submitted to a rRT-PCR assay from Centers of Disease Control and Prevention (CDC) which amplifies the VP1 region of the poliovirus genome. Two assays were successively run: Real-time ITD Assay using six set of primers: Pan Enterovirus, Pan Poliovirus, Poliovirus serotype 1, 2 and 3, and multiplexed targeting Sabin 1, 2 and 3); Real-time VDPV Screening Assay (targeting known Sabin 1, 2 and 3 regions involved in Sabin viruses reversion to neurovirulence) Poliovirus isolates showing discrepancies between rRT-PCR ITD test and VDPV screening may represent Vaccine-Derived Polioviruses (VDPVs) i.e. drifted viruses from parent OPV strains. They were further characterized by sequencing of the full VP1 region of the genome [34].

### Identification of non-polio enteroviruses isolates

Micro-neutralization tests with Lim Benyesh-Melnick (LB-M) pools of mixed equine antisera specific for common EV serotypes were used for identification of selected non-polio enteroviruses according to WHO standard protocols. Viruses that are non typable by microneutralization were classified as untypable.

### Intratypic differentiation of poliovirus strains by enzyme-linked immunosorbent assay

As polioviruses mixture is frequent in sewage specimens and that homotypic mixtures cannot be excluded consequently, All type 1 and type 3 poliovirus isolates obtained after microneutralisation were tested on Elisa as described according to Who standards protocols [35].

## Results

**Figure 2:**
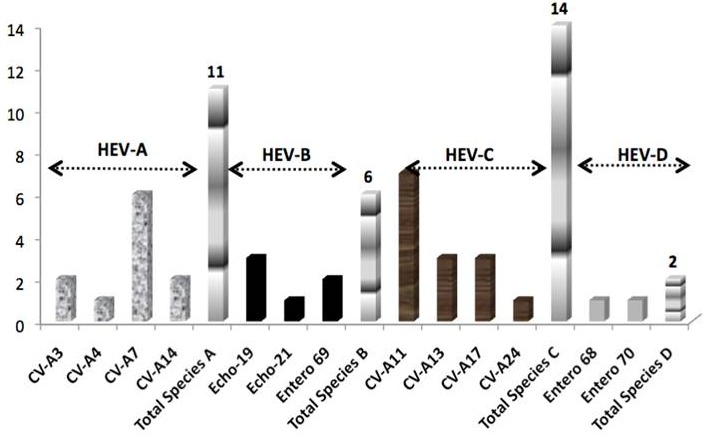
Identification of NPEVs isolates

From the 271 samples of raw sewage collected in all sites, 216 (79, 7%) were positive at least on one cell line. CPE on the 3 cell lines was observed in 43 samples (15, 9%), whereas 73 samples (27%) showed CPE in only one cell line (49 on RD only and 24 on Hep-2c only). Polioviruses (PV) were detected in 93 samples (34, 3%) and all came out as Sabin-Like strains (N = 110) after intratypic differentiation by rRT-PCR. Repartition within the three serotypes was as follows: 29 PV1 (31.18%), 58 PV2 (62.37%), and 28 PV3 (30, 11%) (Of note, 22 samples came out as mixtures of Sabin Like Polioviruses (Table 1). Non polio enteroviruses only were isolated from 116 samples i.e. 42, 8% of the collected samples. Twenty three poliovirus (PV) Sabin Like 2 and 1 PV Sabin Like 3 polioviruses showed discordances between the 2 PCR assays used. They were sequenced in VP1 and compared to Sabin viruses in order to see if they met VDPV definition i.e. > 5 nucleotides difference for PV2 and > 9 nucleotides difference for PV1 and PV3 VDPV. The nucleotides changes were not significant between the virus sent for sequencing and the homotypic Sabin virus. None vaccine-derived poliovirus (VDPV) was detected in our study. No double reactive result, i.e. homotypic mixture of wild and sabin viruses, was noticed. Out of the 116 isolates negative on L20B and positive on Hep2 and/or RD cell lines, 49 non polio enteroviruses (42,2%), confirmed by Pan EV RT-PCR were randomly selected for identification by microneutralization tests. From the 33 serotypable EVs, 14 different serotypes of non polio enteroviruses were detected (Figure 2): 11 HEV-A (33, 3%), 6 HEV- B (18.2%), 14 HEV-C (42.4%) and 2 HEV-D (6%). Of note, 2 parechoviruses were identified. Fourteen non polio enteroviruses (28, 6% of the selected NPEVs) were non typable by antisera pools. No seasonal patterns were noted, particularly for 2008 with a year round environmental surveillance. 3 peaks are noted for February, June and October.

**Table 1:** Polioviruses isolated from sewage samples

		Poliovirus isolated (no wild poliovirus)						
	Samples tested	Single Type			Bivalent mixture			Trivalent mixture
Sites		1	2	3	1 + 2	1 + 3	2 + 3	1 + 2 + 3
Camberene	82	5	21	4	3	3	1	0
Ouakam	28	2	1	0	0	0	1	0
Pikine	49	4	7	5	3	1	1	0
Soumbedioune	21	2	0	0	0	0	0	0
University	71	2	12	5	1	2	5	0
Virage	20	0	1	0	1	0	0	0
Total	271	15	42	14	8	6	8	0

## Discussion

A wide variety of EVs belonging to the four human enterovirus species were found in collected samples, with an isolation rate (79, 7%) consistent to what was described in different studies [36, 37]. Precedent surveys showed a total isolation rate of 65,15% in Yopougon (Abidjan) [38]. In Iran, Enteroviruses and Non-Polio Enteroviruses were respectively isolated from 49 (56.98%) and 46 (53.49%) of specimens [39]. In an another study in three Iranian provinces (Sistan & Baluchestan, Tehran and Fars) in 2009, authors isolated from specimens respectively 49 enteroviruses (56.98%), 38(60.32%) and 11(22.92%) [40]. In Finland, 72 enteroviruses (77, 4%) were identified positives out of 93 studied from sewage. HEV-C species was predominant in our study (42, 4%), followed by HEV-A (33, 3%), HEV-B (18,2%) and HEV-D (6%) [41].

In Singapore, a study detected same prevalence in environmental surveillance with76,5% of HEV-C (coxsackieviruses A-1, A-11, A-17, A22 and A-24), followed by HEV-A species (64,7%) with enterovirus 71 and 89, coxsackievirus A2, A5 and A16. HEV-B species represented 41,2%. HEV-D species were no detected in this study [42]. In our study, Coxsackieviruses represented the majority (75% of serotyped viruses) with coxsackie A-7 and coxsackie A-11 being the more represented. Some of these EVs are associated with diseases in human; EV68 who have both properties of enteroviruses and rhinoviruses is related to severe respiratory diseases [43, 44], EV 70 and Cox-A24 are the major etiological agents involved in acute hemorrhagic conjunctivitis (AHC) outbreaks worldwide, the first AHC outbreak was described in 1969 in Ghana, West Africa, and was called Apollo disease. Since this first reports, the infection has been described in numerous other countries, (China, India, Egypt, Cuba, Singapore, Taiwan, Japan, Pakistan, Thailand, United States, etc.) Massive outbreaks of AHC periodically occur in tropical areas and involve large populations [45–48] EV-19 EV-21 and are implicated in congenital and perinatal infection and recently associated to acute flaccid paralysis and fulminant hepatitis [49, 50]. CV-A3, CV-A4, CV-A11, CV-A13 and CV-A17 are associated with herpangina and Hand Foot and Mouth Disease. 2 echoviruses 23 (now reclassified as parechovirus) were identified; Those viruses are recognized to cause mild gastro intestinal and respiratory illness, myocarditis and encephalitis mainly in children less than 5 years old [51, 52].

In Iran, previous study in environment showed a wide variety of Enterovirus Non Polio with EV-11 (31.52%), CV-B (27.58%), EV-7 (17.73%) and EV-4 (21.67%) [53]. 28, 6% of Enterovirus Non Polio were non typable in our study by microneutralization using two pools of antisera, 77% were non typable by using one pool of anti sera in Iranian observation [53]. We can observe large fluctuations in circulation over time. In temperate climates, enteroviral infections are most common in the summer and early fall, but seasonal patterns are less evident in tropical areas where circulation trends are year round. In a Ghanaian study, the peaks of NPEVs’ infection were between July and August and in a lower level from November to January.

## Conclusion

This study is the first one depicting enteroviruses detection in sewage specimens in Senegal. Even though some disease-specific programmes, like the polio eradication programme, has proven to be efficient in detecting EVs circulating within the population, very little is known about implications of EVs in a vast array of diseases in tropical settings. Results gathered from environmental surveillance of EVs can help targeting specific disease-associated pathogens in the community within the catchment area of sampling sites. Most importantly, poliovirus were found in approximately one third of the samples, proving that the sampling sites can be successfully used to sensitively monitor poliovirus (wild, VDPV and Sabin) circulation within a population pre- and post-eradication. Moreover, this will add value on AFP surveillance, not only in Senegal but also in west Africa as Dakar is an important hub for travelers/migrants from other west-african countries. Moreover, 42% of EVs that were identified belongs to HEV-C species that are known to recombine with PV and increase the probability of VDPV emergence. [54] On another perspective, enteroviruses may be a source of contamination in community through utilization of improperly treated wastewater for drinking, bathing, and irrigating especially in developing countries where resources in water are limited. This study set the basis for an agreement between the National Polio laboratory and ONAS (Office National de l'Assainissement du Senegal), the company managing sewage treatment plants, to use this protocol for (i) assessing effectiveness of water treatment that aim to ride off viral agents and (ii) monitoring of treated water that is further used for agricultural purposes.
